# Cobalamin c deficiency associated with antifactor h antibody-associated hemolytic uremic syndrome in a young adult

**DOI:** 10.1186/s12882-020-01748-2

**Published:** 2020-03-12

**Authors:** C. Philipponnet, J. Desenclos, M. Brailova, J. Aniort, J. L. Kemeny, C. Deville, V. Fremeaux-Bacchi, B. Souweine, A. E. Heng

**Affiliations:** 1 Nephrology, Dialysis and Transplantation Department, CHU Clermont Ferrand, University Clermont Auvergne, Clermont Ferrand, France; 2 Biochemistry Department, CHU Clermont Ferrand, University Clermont Auvergne, Clermont Ferrand, France; 3 Anatomy and Pathology Department, CHU Clermont Ferrand, University Clermont Auvergne, Clermont Ferrand, France; 4 Assistance Publique-Hopitaux de Paris; Laboratory of Immunology, Georges Pompidou Hospital, Paris, France; 5 Médecine intensive et réanimation, CHU Clermont Ferrand, University Clermont Auvergne, Clermont Ferrand, France

**Keywords:** Thrombotic microangiopathy, Cobalamin C (Cbl C) disease, Atypical hemolytic uremic syndrome, Anti-factor H antibody

## Abstract

**Background:**

Thrombotic microangiopathy (TMA) syndromes are characterized by the association of hemolytic anemia, thrombocytopenia and organ injury due to arteriolar and capillary thrombosis.

**Case presentation:**

We report the first case of adult onset cobalamin C (Cbl C) disease associated with anti-factor H antibody-associated hemolytic uremic syndrome (HUS).

A 19-year-old woman was admitted to the nephrology department owing to acute kidney failure, proteinuria, and hemolytic anemia with schizocytes.

TMA was diagnosed and plasma exchanges were started in emergency.

Exhaustive analyses showed 1) circulating anti factor H antibody and 2) hyperhomocysteinemia, hypomethioninemia and high levels of methylmalonic aciduria pointing towards Clb C disease. Cbl C disease has been confirmed by methylmalonic aciduria and homocystinuria type C protein gene sequencing revealing two heterozygous pathogenic variants.

The kidney biopsy showed 1) intraglomerular and intravascular thrombi 2) noticeable thickening of the capillary wall with a duplication aspect of the glomerular basement membrane and a glomerular capillary wall IgM associated with Cbl C disease related TMA.

We initiated treatment including hydroxycobalamin, folinic acid, betaine and levocarnitine and Eculizumab. Rituximab infusions were performed allowing a high decrease in anti-factor H antibody rate.

Six month after the disease onset, Eculizumab was weaning and vitaminotherapy continued. Outcome was favorable with a dramatic improvement in kidney function.

**Conclusion:**

TMA with renal involvement can have a complex combination of risk factors including anti-FH autoantibody in the presence of cblC deficiency.

## Background

Thrombotic microangiopathy (TMA) syndromes are characterized by the association of hemolytic anemia, thrombocytopenia and organ injury due to arteriolar and capillary thrombosis [1]. TMA is a life-threatening condition requiring emergency care and etiologic diagnosis is of paramount importance for directing treatment and improving patient and renal survival [1]. In adults, the causes of TMA include Shiga toxin-related hemolytic (STEC) uremic syndrome (HUS), a disintegrin and metalloproteinase with a thrombospondin type 1 motif, member 13 (ADAMTS13)-acquired deficiency and atypical HUS (aHUS) secondary to complement alternative pathway dysregulation. However, aHUS can result from other conditions including malignant hypertension, malignancy, adverse drug effect, systemic disease, infections, pregnancy, and transplantation [1]. In newborns, recessive mutations in diacylglycerol kinase ɛ (DGKE) cause aHUS, and in children, cobalamin C (cblC) deficiency is a rare genetic disease that can also lead to aHUS [1]. We describe a case of adult-onset cblC disease associated with anti-factor H antibody-associated HUS and kidney biopsy pathologic findings. To the best of our knowledge such a combination has not been previously reported. The patient has provided written consent for publication of the case.

## Case presentation

A 19-year-old woman was admitted to the medical intensive care unit for acute kidney failure associated with severe neurologic impairment. She had no relevant past medical history and her familial history was unremarkable. She presented with asthenia and dyspnea of several weeks duration. Clinical examination showed high blood pressure of 170/90 mmHg and lower limb edema. No systemic sign was detected and no gastrointestinal disorder reported. The initial laboratory test results were as follows: serum creatinine 246 μmol/L, hemoglobin 8,9 g/dL, platelets 151 G/L, lactate deshydrogenase 700 U/L (normal < 246), haptoglobin < 0,08 g/L, with schistocytes on peripheral blood smear, proteinuria 7.6 g/g of creatinine, and hematuria 348.10^3^/mL. Direct antiglobulin and pregnancy tests were negative. Transthoracic echocardiography showed preserved left ventricular function and renal ultrasonography excluded an obstructive cause of acute kidney injury. A diagnosis of TMA was considered. Samples for an extensive diagnostic work-up were obtained and plasma exchanges were started in emergency. On day 2, after thrombotic thrombocytopenic purpura had been excluded on the basis of normal serum ADAMTS13 values (101%), plasma exchanges were stopped and eculizumab (900 mg per week) was initiated.

The diagnosis of STEC-HUS was ruled out on the basis of negative Shiga toxin tests. Antibody testing for hepatitis B, hepatitis C, human immunodeficiency virus, antinuclear antibody, lupus anticoagulant, antiphospholipid, anti-deoxyribonucleic acid and neutrophil cytoplasmic antigen antibodies was negative. Exploration of the alternative complement pathway including measurement of serum levels of hemolytic complement CH50, complement C3, C4, Factor H (CFH) and factor I was normal, 109%, 697 mg/L, 153 mg/L, 106, and 95%, respectively. No mutations in C3, MCP, factors B, H, and I genes were identified. Antibody testing for CFH was positive: 7450 UA/ml; reference range < 100 AU/mL. The serum level of homocysteine was increased: 285 μmol/L for reference values ranging between 3.2 and 10.7. The serum levels of vitamin B12 and folate were normal.

**Fig. 1:**
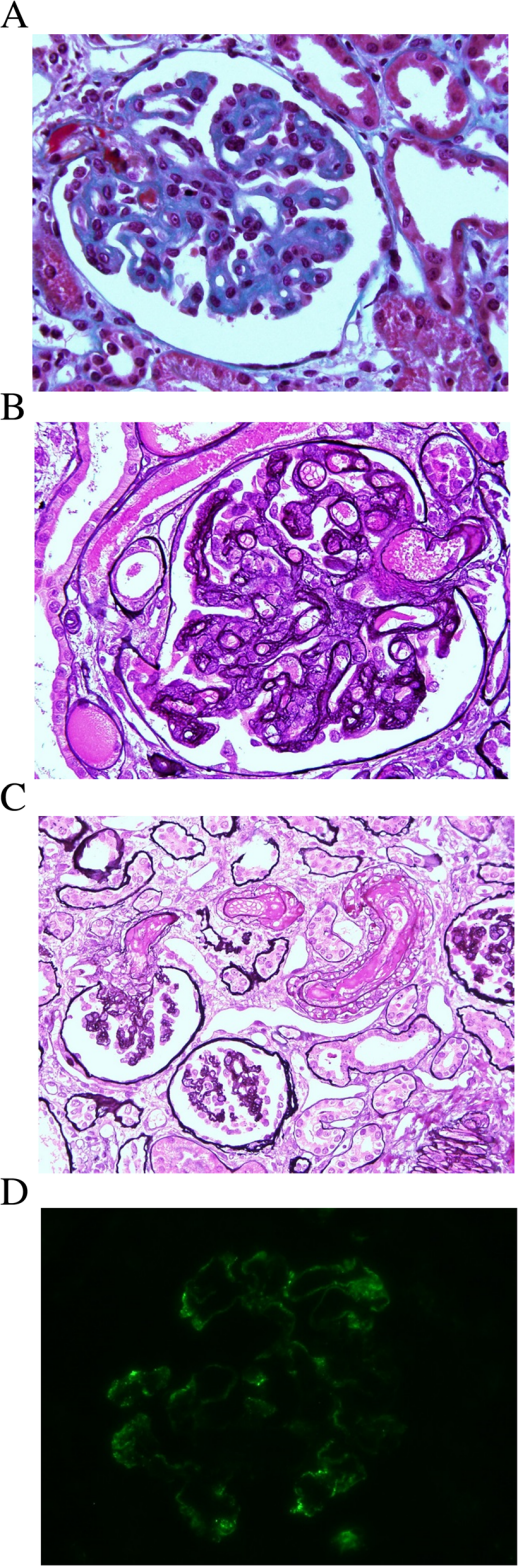
Kidney histology. **a** Light microscopy, Masson’s trichrome staining, × 40. There was a diffuse augmentation of mesangial material and mesangial hypercellularity in all glomeruli, and a thickening of the glomerular basement membranes. Thrombi were present in the intraglomerular capillaries and in vessels of the intra juxtaglomerular apparatus. **b** Light microscopy, silver staining, × 60. Thickening of the capillary wall with a duplication aspect of the glomerular basement membrane. **c** Light microscopy, silver staining, × 40. Thrombi were present in vessels of the intra juxtaglomerular apparatus and in the arterioles. **d** Immunofluorescence, IgM staining, × 60. Mild glomerular capillary wall IgM deposits

A kidney biopsy performed on day 2 obtained 17 glomeruli and showed mesangial sclerosis, noticeable thickening of the capillary wall with a duplication aspect of the glomerular basement membrane (GBM) and the presence of intraglomerular and intravascular thrombi (Fig. 1). No tubular or interstitial damage was observed. Immunofluorescence studies showed mild glomerular capillary wall IgM and C3 deposits and were negative for IgG and IgA. The duplication of GBM, the presence of positive IgM deposits, the worsening of kidney function with increased serum creatinine levels of 700 μmol/L despite eculizumab treatment, and high serum levels of homocystein suggested an inborn error of intracellular cobalamin metabolism and prompted complementary tests. Results of plasma and urinary amino acid chromatography indicated low levels of plasma and urine methionine: 12 μmol/L (reference range of 21–35), < 1 (reference range of 2–16 μmol/mmol of creatinine), respectively. Results of gas chromatography-mass spectrometry of urinary organic acid indicated high levels of methylmalonic aciduria: 247 mmol/mol of creatinine (normal < 4 μmol/l). Both results guided etiological diagnosis toward cblC disease.

**Fig. 2:**
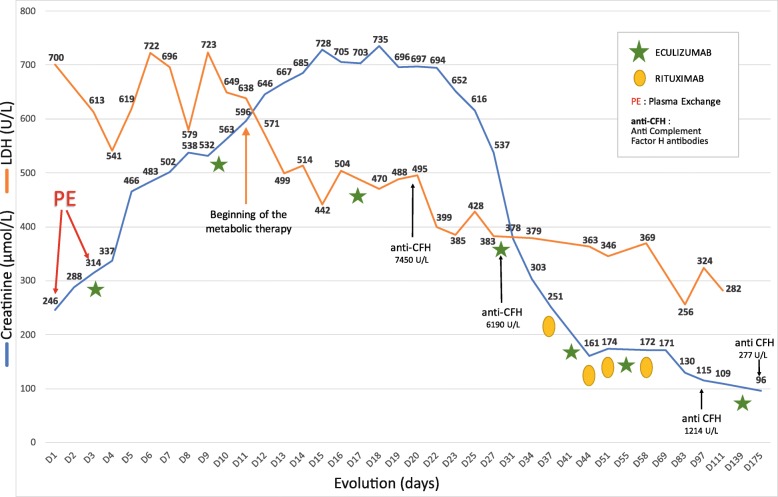
Patient outcome

Continuous vitamin supplementation with hydroxycobalamin 1 mg/d by intramuscular route, folinic acid 10 mg/d, betaine 12 g/d and levocarnitine 3 g/d was started. In the following days, kidney function dramatically improved and hemolysis resolved (Fig. 2). Normal values of serum homocysteine and of plasma methylmalonic acid were achieved within the following month. Rituximab infusion at 375 mg/m2 was administered once a week for 4 weeks and resulted in a dramatic decrease in serum anti-factor H antibody (< 1000 UA/ml) and led to weaning from eculizumab. Six months later, the patient had no signs of hemolysis, a serum creatinine value of 75 μmol/L (eGFR 100 mL/min CKD EPI) and serum anti-factor H antibody were negative.

Methylmalonic aciduria and homocystinuria type C (MMACHC) gene sequencing showed two heterozygous pathogenic variants in our patient, c.556G > A (p.Arg189His) and c.271dupA (p.Arg91Lysfs*4), with c.271dupA (p.Arg91Lysfs*14) variant identified in her father and c.566G > A (p.Arg189His) in her mother. cblC deficiency associated with anti-factor H antibody-associated hemolytic uremic syndrome was ultimately diagnosed.

## Discussion and conclusions

Cb1C deficiency is a rare autosomal recessive disorder caused by mutations in the MMACHC gene [2]. It is the most common inborn error of vitamin B12 metabolism.

The defective MMACHC protein causes decreased levels of adenosylcobalamin (AdoCbl) and methylcobalamin (MeCbl), cofactors for the enzymes methylmalonyl-CoA mutase and methionine synthase [3]. MeCbl is required for the intracytoplasmic methylation of homocysteine in methionine by the methionine synthase. AdoCbl acts as a cofactor for the methylmalonyl coenzyme A mutase during the transformation of L-methylmalonyl-CoA into succinyl-CoA. Hence, the biological hallmarks of cblC disease are hyperhomocysteinemia with low methioninemia and methylmalonic aciduria (MMA) [3]. It is strongly recommended that in case of suspicion of cblC deficiency to start investigations by an assay of the total plasma homocysteine. If total homocysteine is elevated, plasma and urine samples should be collected for determination of MMA, methionine, folate and vitamin B12 before initiating emergency treatment [4].

Currently, 87 mutations are identified in the MMACHC gene, of which c.271dupA mutation is the most frequently encountered. Heterozygous patients seem to have late onset disease. The phenotype appears to be determined by the less deleterious mutations [5]. Our patient, like other patients with late onset TMA in cblC deficiency, is heterozygous with c.271dupA mutation and missense mutation (c.556G > A in our case) [5, 6].

In most instances cblC disease occurs during the first year of life with severe phenotype including neurological involvement, such as microcephaly, seizure, developmental delay, ataxia and hypotonia, megaloblastic anemia and/or cardiac, renal and ocular disorders.

Kidney failure in cblC deficiency is due to TMA. The etiology of endothelial damage in this setting remains largely unknown [2, 3]. Hyperhomocysteinemia induces vascular endothelial toxicity and alterations of the antithrombotic properties of the endothelium. Interestingly, isolated hyperhomocysteinemia (i.e. without hypomethioninemia and/or methylmalonic acidemia) has not been reported to cause any specific renal disease apart from thromboembolism. Possibly, additional biochemical abnormalities such as methylmalonic acidemia and/or methionine deficiency are required to develop TMA [7]. Recent studies have also shown the role of oxidative stress (heat shock proteins, ubiquitins and proteins involved in the glutathione pathway). The increased production of reactive oxygen species triggers endoplasmic reticulum stress and apoptosis [7].

Late-onset disease is seen in adolescents and young adults. It is often a challenging diagnosis because kidney failure with hemolysis is the sole sign of the disease and thrombopenia can be absent in this specific setting of TMA. Only a few cases of late-onset cblC deficiency- related HUS have been reported in the literature [6, 8].

A recent report described a series of seven patients aged from 6 to 26 years with histologically proven renal TMA in the setting of late-onset cblC deficiency [8]. Histological analysis showed glomerular and arteriolar TMA in all patients with intravascular and intraglomerular thrombi [8]. The histology of the 7 patients was compared with that of 16 controls with cblC deficiency independent TMA: a vacuolated aspect of the glomerular basement membrane and intense deposits of glomerular capillary wall IgM were, as in our patient, more marked in the case of cblC deficiency [8].

Treatment of cblC deficiency is based on supplementation containing high doses of vitamin B12, betain and folic acid [4]. This supplementation dramatically improved renal function in our patient, as in other documented cases.

The peculiarity of our patient was the association of cblC deficiency with the presence of circulating anti-factor H antibody.

There have been two previously reported cases of cblC deficiency and complement alternative pathway dysfunction. The first involved a 6-year-old girl with thrombotic microangiopathy caused by the association of cblC deficiency and heterozygous factor H mutation [9]. She was treated with plasma exchanges and vitamin therapy, which allowed dialysis weaning but was followed by persistent chronic renal failure. The second patient was a 6-month-old male infant who had microangiopathy caused by cblC deficiency [10]. Despite vitamin therapy, evolution was not favorable with hemodialysis requirement. The infant’s C3 level was decreased and an alternative complement pathway-associated dysfunction was suspected. Eculizumab was initiated despite there being no abnormalities of the alternative complement pathway detected and dialysis weaning was achieved [10].

To our knowledge, this case report of cblC deficiency associated with factor H (FH) antibody is the first to be documented.

Anti-FH–associated atypical hemolytic uremic syndrome (aHUS) occurs predominantly in childhood [11]. A strong association has been observed between anti-FH autoantibodies and a homozygous deletion of CFHR1 and CFHR3, which encode complement FH–related proteins 1 and 3 [12]. In our patient, no sign of complement activation was observed in plasma, the plasma CFH antigenic level was normal and no abnormalities were found in the genes CFH, CFI, CFB, MCP, and C3. The patient carried two copies of the CFHR1-CFHR3 genes.

Abnormalities of the alternative complement pathway could coexist with cblC deficiency via a dual mechanism.

In our patient, outcome dramatically improved with vitamin therapy and eculizumab. Rituximab therapy has been prescribed for the treatment of anti-factor H antibody [13, 14]. In our patient, this strategy allowed eculizumab weaning after anti-factor H antibody levels were < 1000 UA/mL.

cblC deficiency is a cause of microangiopathy even in the young adult and can be revealed by isolated renal failure in the absence of thrombopenia. Its incidence is probably underestimated and homocysteine should be measured in patients with aHUS [15]. TMA with renal involvement can have a complex combination of risk factors including anti-FH autoantibody in the presence of cblC deficiency.

## Data Availability

Not applicable.

## Abbreviations

ADAMTS13: A thrombospondin type 1 motif, member 13
AdoCbl: Adenosylcobalamin
aHUS: Atypical hemolytic uremic syndrome
Cbl C: Cobalamin C
CFH: Factor H
GBM: Glomerular basement membrane
HUS: Hemolytic uremic syndrome
MeCbl: Methylcobalamin
MMA: Methylmalonic aciduria
MMACHC: Methylmalonic aciduria and homocystinuria type C
STEC: Shiga toxin-related hemolytic
TMA: Thrombotic microangiopathy
